# Prevalence and Molecular Characterization of Extended-Spectrum β-Lactamase Producing *Enterobacterales* in Healthy Community Dogs in Israel

**DOI:** 10.3390/antibiotics11081069

**Published:** 2022-08-07

**Authors:** Anat Shnaiderman-Torban, Shiri Navon-Venezia, Hadar Baron, Wiessam Abu-Ahmad, Haya Arielly, Gal Zizelski Valenci, Israel Nissan, Yossi Paitan, Amir Steinman

**Affiliations:** 1 Koret School of Veterinary Medicine, The Robert H. Smith Faculty of Agriculture, Food and Environment, The Hebrew University of Jerusalem, Rehovot 7610001, Israel; 2 Molecular Biology Department, Ariel University, Ariel 4076414, Israel; 3 The Dr. Miriam and Sheldon G. Adelson School of Medicine, Ariel University, Ariel 4076414, Israel; 4 Hadassah Braun School of Public Health and Community Medicine, Hebrew University of Jerusalem, Jerusalem 9112102, Israel; 5 Clinical Microbiology Lab, Meir Medical Center, Kfar Saba 4428164, Israel; 6 National Public Health Laboratory, Public Health Services, Ministry of Health, Tel Aviv 6810416, Israel; 7 Ilex Labs, Ilex Medical Ltd., 7 Hatnufa St., Petach Yikva 2951025, Israel

**Keywords:** ESBL, *Enterobacterales*, dogs, coprophagia, antibiotic resistance

## Abstract

Background: antimicrobial resistance is a global problem in human and veterinary medicine. We aimed to investigate the extended spectrum β-lactamase-producing *Enterobacterales* (ESBL-PE) gut colonization in healthy community dogs in Israel. Methods: Rectal swabs were sampled from 145 healthy dogs, enriched, plated on selective plates, sub-cultured to obtain pure cultures, and ESBL production was confirmed. Bacterial species and antibiotic susceptibility profiles were identified. WGS was performed on all of the ESBL-PE isolates and their resistomes were identified in silico. Owners’ questionnaires were collected for risk factor analysis. Results: ESBL-PE gut colonization rate was 6.2% (*n* = 9/145, 95% CI 2.9–11.5). Overall, ten isolates were detected (one dog had two isolates); the main species was *Escherichia coli* (eight isolates), belonging to diverse phylogenetic groups—B1, A and C. Two isolates were identified as *Citrobacter braakii*, and *C. portucalensis*. A phylogenetic analysis indicated that all of the isolates were genetically unrelated and sporadic. The isolates possessed diverse ESBL genes and antibiotic-resistance gene content, suggesting independent ESBL spread. In a multivariable risk factor analysis, coprophagia was identified as a risk factor for ESBL-PE gut colonization (*p* = 0.048, aOR = 4.408, 95% CI 1.014–19.169). Conclusions: healthy community dogs may be colonized with ESBL-PE MDR strains, some of which were previously reported in humans, that carry wide and diverse resistomes and may serve as a possible source for AMR.

## 1. Introduction

Antimicrobial resistance is recognized as a major, global, interdisciplinary health challenge, with significant human society costs due to increased mortality, morbidity and use of health care resources [1]. Extended-spectrum β-lactamases (ESBL) are enzymes produced by Gram-negative bacteria from the *Enterobacterales* order, which hydrolyze penicillins, extended-spectrum cephalosporins and monobactams, and are inhibited by β-lactamase inhibitors, such as clavulanic acid and tazobactam [2]. The plasmids that encode ESBL genes often encode additional antimicrobial resistance (AMR) genes that confer resistance to other antibiotics, including aminoglycosides, sulfonamides and quinolones. Therefore, ESBL-producing *Enterobacterales* (ESBL-PE) are commonly multidrug-resistant (MDR), which poses a particular challenge for the treatment of infections [3].

*Enterobacterales* is a heterogeneous group of gammaproteobacteria, widely distributed in nature, and many of their species are gut-commensal or enteric pathogens in both humans and animals [4]. The intestinal tract is an important reservoir for nosocomial pathogens, including *Enterobacterales* [5], and ESBL-PE gut colonization was identified in humans as a risk factor for subsequent infections [6]. The infections caused by ESBL-PE in humans are associated with a delay in the initiation of appropriate antibiotic therapy, with increased costs and with worse clinical outcomes [7].

ESBL-PE were reported as gut-colonizing in farm animals [8,9,10], wild animals [11,12,13] and companion animals, including dogs, worldwide [14,15,16]. In the context of human health, dogs may have a special role due to their close contacts with humans and, indeed, the co-carriage of ESBL-PE in dogs and humans in the same households was demonstrated [17,18,19]. The information regarding the prevalence and molecular characterization of ESBL-P *Escherichia coli* genes in dogs and cats was recently reviewed [20]. However, the data on ESBL-PE in Middle-Eastern countries is still limited [21,22,23]. In Israel, gut colonization with ESBL-PE was reported in cattle [10], horses [24], petting-zoo animals [25] and small companion animals (dogs and cats) on hospital admission [26], but data regarding healthy household dogs is still scarce. Therefore, we aimed to investigate the prevalence, molecular characteristics and the risk factors associated with ESBL-PE gut colonization in healthy dogs in the community in Israel. 

## 2. Results

### 2.1. Characterization of Study Population and Prevalence of ESBL-PE Gut Colonization

Overall, 145 dogs were sampled (February 2016 to January 2018), originating from 112 private households (34 of them were sampled during a participation in a weekly training group), and 33 that originated from two breeding farms (27 dogs from one farm and six dogs from a second farm). The median age was 2.6 years (range: one month- 12.5 years). The sex was recorded for 138 dogs, of which 29.0% (*n* = 40) were intact females, 34.8% (*n* = 48) were spayed females, 14.5% (*n* = 20) were males and 21.7% (*n* = 30) were castrated males. The data regarding the dogs’ breeds were available for 142 dogs, and included 24 dogs’ breeds, in addition to mixed-breed dogs. Sixty-four (45.1%) of the dogs were mixed breeds and the most common breeds included Cane Corso (12.0%, *n* = 17) and Belgian Malinois and Border collie (6.3%, *n* = 9 each). The gut colonization rate of ESBL-PE was 6.2% (*n* = 9/145, 95% CI 2.9–11.5) and was 5.5% (*n* = 8/145, 95% CI 2.4–10.6) for ESBL *E. coli*. All of the colonized dogs originated from private households.

### 2.2. Antibiotic Resistance Profiles of the ESBL-PE Isolates

Most of the isolates were resistant to trimethoprim-sulfamethoxazole (60%, *n* = 6/10) and half of the isolates were resistant to amoxicillin-clavulanate and to ciprofloxacin (*n* = 50%, *n* = 5/10). A minority of the isolates were resistant to the aminoglycosides amikacin (10%, *n* = 1/10) and gentamicin (20%, *n* = 2/10). All of the isolates were susceptible to nitrofurantoin and carbapenems and resistant to cephalosporines. Three of the isolates (30%) were identified as MDR.

### 2.3. Distribution of ESBL-PE Species and Whole Genome Sequencing Taxonomic Classification

**Figure 1:**
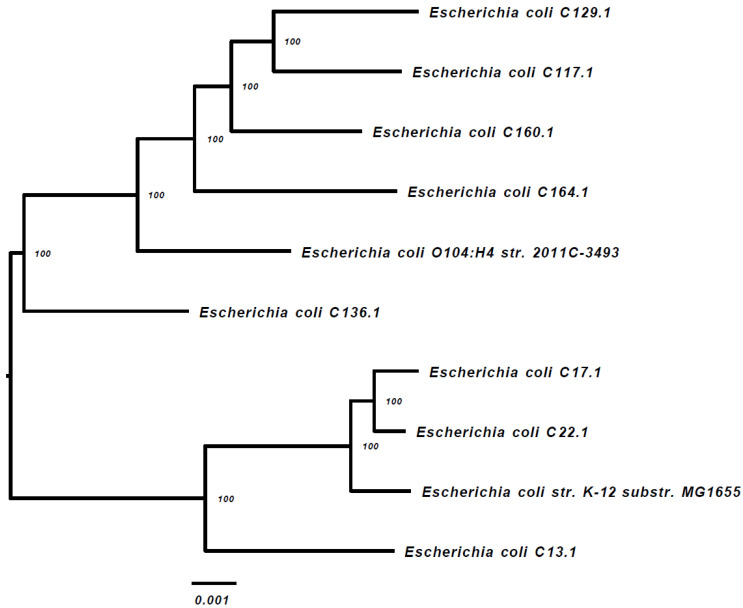
WGS-based phylogenetic tree of the 8 *E. coli* gut isolates (including two reference strains).

Ten of the ESBL-PE isolates were subjected to WGS (one dog had two isolates), including *E. coli* (*n* = 8 isolates), *Citrobacter braakii* and *C. portucalensis* (one isolate each). The alignment of the 1000 proteins and coding DNA shared by all of the isolates and appearing in a single copy, indicates that all of the isolates are sporadic (Figure 1). Strains C17.1 and C22.1 were phylogenetically close (Figure 1) and belong to the same sequence type (ST) (Table 1).

The Phylogenetic Tree Building Service of the PATRIC v3.5.36 platform [27] was used to create the tree, as described in the Materials and Methods section. The *E. coli* strains k-12 substrain MG16555 and *E. coli* O104:H4 str. 2011C-3493 were used as the references.

### 2.4. ESBL-PE Resistome

Table 2 summarizes the antibiotic resistance genes, mainly acquired, identified in silico by the NDARO database through the PATRIC server [27], from the WGS data of the ten isolates. Table 2 also presents the plasmid replicons identified by the MobileElementFinder web tool [28]. A comprehensive analysis of all of the genes that may contribute to the isolate resistance was performed using CARD, by utilizing the Resistance Gene Identifier (RGI) function (Table S1, Supplementary Materials). In addition, Table S2 (Supplementary Materials) includes comprehensive information about the mobile genetic elements (MGEs), detected by the MobileElementFinder web tool [28] at the Center for Genomic Epidemiology [29].

All of the ESBL-PE resistomes included at least one β-Lactamase gene, with the majority of the isolates harboring three β-Lactamase genes (70%), and at least one additional antibiotic resistance gene, conferring resistance to different antibiotic class (90%). In most of the isolates, the resistance genes are located on the plasmids (Table 2). Based on blastn search, the plasmid that originated from C17.1 and C164.1 was probably acquired from *Klebsiella pneumonia*. Isolates C129.1 and C129.2 possess plasmids that are similar (but not identical) to the pSS44 plasmid (Table 2). Isolate C23.2 genome harbors only two resistance genes; both located on the chromosome, the AmpC *bla*CMY-34 gene (for β-Lactamases) and a QnrB9 gene (for Quinolones).

### 2.5. Risk Factor Analysis for ESBL-PE Gut Colonization

In a univariable analysis (Table 3), coprophagia (*p* = 0.024) was associated with ESBL-PE gut colonization. The variables ‘age’ (*p* = 0.179) and ‘supervised walks’ (*p* = 0.165) were included in the multivariable model (*p* ≤ 0.2). In the multivariable analysis (Table 4), coprophagia was identified as a risk factor for ESBL-PE gut colonization (*p* = 0.048, aOR = 4.408, 95% CI 1.014–19.169).

## 3. Discussion

The global prevalence and molecular characterization of ESBL-P *E. coli* in dogs and cats was recently reviewed, and was 6.87% (95% CI: 4.46–10.45%) [20]. The prevalence varied between different continents, ranging from 0.63% (95% CI: 0.02–15.34%) (Oceania) to 16.56% (95% CI: 5.26–42.45%) (Asia) [20]. Limited information is available regarding the prevalence of ESBL among dogs in Middle Eastern countries. In a study, that was conducted in 2014 in Turkey, 16.8% of the isolates from companion dogs exhibited the ESBL phenotype [23]. This was similar to what was found in 49 healthy dogs in Greece (20.4%) [22] and in 68 dogs in Egypt (22%) [21]. In this study, the ESBL-PE (*E. coli* and *Citrobacter* spp.) gut colonization rate in healthy dogs in the Israeli community was 6.2% (*n* = 9/145, 95% CI 2.9–11.5), lower than was previously found in neighboring countries. The ESBL-P *E. coli* gut colonization rate was 5.5%, which is similar to the average global prevalence [20]. This prevalence is significantly lower compared to the colonization rates, identified previously in Israel, in dogs on hospital admission, 22.8% (*n* = 43/189, 95% CI 17–29.4, *p* < 0.0001) [26], and in healthy farm horses, 20.8% (*n* = 40/192, 95% CI 15.3–27.3, *p* < 0.0001) [24], during the same years. The dogs on hospital admission differ from the healthy dogs in the community, since many of them were treated earlier by private veterinarians, and only later referred to the hospital. This may also partially explain the higher ESBL-PE carriage rate found in dogs in Egypt, some of which were sampled during a visit to the university hospital [21]. The higher rate of ESBL-PE colonization in farm horses is similar to what was found in cattle in Israel [10], and may be related to differences in management and living habitat.

The main ESBL-PE species identified in this study was *E. coli*, as was reported previously in canine fecal samples [14,21,26]. Three *E*. *coli* phylogenetic groups were identified, including B1 (four isolates), A (three isolates) and C (one isolate). It was found that human pathogenic *E. coli* strains causing extra intestinal infections mainly belong to group B2 and a lesser extent to group D, whereas the commensal strains belong to group A and B1 [30]. All of the phylogenetic groups (A, B1, B2, C, D, E and F) were found in ESBL-P *E. coli* isolates from America and Europe, whereas the phylogroup C was not detected in Africa, and the phylogroup E was not detected in Asia [20]. The phylogrouping results were further confirmed by a WGS-based phylogenetic tree that demonstrated the sporadic nature of the ESBL-P *E. coli* isolates identified in our study. These genomic findings supports the fact that the dogs’ population in this study were from different independent households.

A WGS-based analysis of the 10 ESBL-PE resistomes identified highly diverse antibiotic-resistance gene (ARGs) combinations carried by the study isolates, including genes conferring resistance to macrolides, tetracycline and chloramphenicol, as well as a diverse content of plasmid-mediated ESBLs and quinolone resistance genes (Table 2). Seven of these 10 isolates appear to be resistant to multiple classes of antibiotics. Although three of the *E. coli* isolates belonged to ST10 (Table 1), the WGS-based phylogenetic tree (Figure 1) did not identify clonality among these three sequenced isolates.

The resistome data expand the phenotypic data (Table 1) and provide the genetic basis for the described resistance. For example, the C164.1 genome encodes for the ESBL gene *bla*CTX-M-3. This isolate was also resistant to amikacin (AMK), gentamycin (GENT) and trimethoprim-sulfamethoxazole (TMS). Indeed, its genome encodes for the genes APH(3′′) and AAC(3)-Ii, APH(6)-Id, which confer resistance to aminoglycosides, as well as for Sul1 and DfrA12, which confer resistance to sulfonamides and trimethoprim, respectively. Isolate C164.1 also contains the gene Mph(E), which confers macrolide resistance. Isolate C117.1 was found to be resistant to quinolones by a VITEC phenotypic test, but in Table 2, no gene for resistance to quinolones appears. CARD—RGI comprehensive analysis (Materials and Methods; Table S1, Supplementary Materials) revealed 14 genes, which may contribute to the resistance of this isolate (*mdtM*, *marA*, *mdtH*, *gadX*, *mdtF*, *mdtE*, *CRP*, *emrR*, *emrA*, *emrB*, *rsmA*, *gyrA*, *parC*, *soxS*). Many of these genes encode for efflux pumps. The D87N and S83L mutations in the *gyrA* and S80I in *parC*, respectively, confer resistance to the fluoroquinolone antibiotics. The example for isolate C117.1 can be expanded for the additional isolates, in which there is a difference between the phenotype and the genotype. Five of the isolates possess phenotypic resistance for amoxicillin clavulanate (Table 1), while three of them do not encode for the AmpC gene (C13.1, C23.2, C129.2). Inducible AmpC is important for amoxicillin clavulanate resistance in *Enterobacter* sp., *Citrobacter freundii*, *Serratia marcescens*, *Morganella morganii*, *Hafnia alvei* and *Providencia stuartii*, which results in intrinsic resistance, but rarely contributes to amoxicillin-clavulanate resistance in *E. coli* [31]. Multiple beta-lactamase are involved in the amoxicillin-clavulanate resistance, mainly from the TEM, AmpC, CMY-2 and the OXA types [32]. A previous study found two different variants of blaTEM-1: blaTEM-1b and blaTEM-1a, being the most dominant beta-lactamases in the AMC-resistant enterobacteria, isolated in Buenos Aires [33]. Amoxicillin-clavulanate resistance is mainly associated with TEM-1 overproduction (mostly in *E. coli*) or co-expressed with OXA-2-like and/or SHV β-lactamases (*K. pneumoniae* and *P. mirabilis*) [33]. Isolate C23.2 encodes for the blaCMY-34 that confers resistance to cephamycin, while isolate C129.2 encodes for the multiple beta-lactamases, blaCTX-M-39, blaTEM-237 and blaCMY-93. We therefore assume that the overexpression of these genes, together with efflux pump activity, as found through CARD—RGI analysis (Materials and Methods; Table S1, Supplementary Materials), confers amoxicillin-clavulanate resistance to these two isolates.

According to a recent review, a total of 171 different ESBL *E. coli* STs were identified in companion animals [20]. In this study, we identified six different STs: ST10 was found in three dogs and was previously reported from dogs and cats in America, Asia, and Europe. *E. coli* ST10 and the closely related STs are frequently recovered from food and human intestinal samples and studies have shown a higher prevalence of the plasmid-carried AMR genes in ST10, including the CTX-M ESBL genes, compared to the other STs [34]. The other five STs were identified in a single dog each. ST46 was previously recovered from dogs in Africa, Asia and in Europe [20], and was also detected in human fecal specimens [35]. ST88 and ST297 were reported previously from dogs in Europe [20]. ST88 was detected before in poultry, meat and human clinical specimens in Europe [36], and ST297 was also detected in human clinical samples [37]. ST602 was reported previously in dogs in Asia and in Europe [20], and is considered to be infrequent in humans but more frequent in animal samples [38]. To the best of our knowledge, ST1586 was not reported previously in dogs, however, it was previously reported from a horse in an equine clinic in the Netherlands [39] and from a horse in the clinic for horses at the Freie Universitat Berlin, Germany [40]. ESBL-P *E. coli* ST1586 was also previously isolated from two cats in the Tyrol, Germany [41].

In previous studies, previous antibiotic treatment, eating raw meat [19,42], walking the dog in a forest and hospitalization or a vet visit in the past four weeks prior to sampling [17], were found as risk factors for gut colonization in healthy dogs. In this study, we report coprophagia as a risk factor for ESBL-PE gut colonization. Coprophagia is a well-known behavior in dogs and was associated with salmonellosis [43] and helminth findings in canine feces, even in the absence of an actual infection [44,45]. Coprophagia was also positively associated with an increased risk of antimicrobial resistance for several antimicrobials in a previous study in dogs and cats in Portugal [46]. Since ESBL-PE are enteric bacteria, finding that coprophagia is a possible risk factor for ESBL-PE gut colonization is logical and this finding may have a significance in the aspects of one health and antimicrobial resistance spread, as dogs may ingest contaminated feces and then secrete ESBL-PE to the environment. 

The limitations of the study include the convenience sampling of a relatively small sample size, the lack of sampling in the dogs’ environment and the cross-sectional study design, and the use of a sampling point for the prevalence of gut colonization. Expanding the sample size and sampling additional private households, breeding farms and shelters may shed light on the complex epidemiology and spread of ESBL-PE gut colonization. Sampling the dogs’ environments, including additional animals and humans, can also reveal additional details regarding antimicrobial sources and spread. A longitudinal study, as opposed to a point prevalence study, may explain the spread mechanisms, and reveal additional risk factors for ESBL-PE shedding and acquisition.

## 4. Materials and Methods

### 4.1. Study Design and Sampling Methods

This prospective study was performed in canine training groups, two canine breeding facilities and in private households in Israel. The study was approved by the Internal Research Review Committee of the KSVM-VTH (Reference number: KSVM-VTH/23_2015). The rectal swabs were collected from the dogs with the owners’ consent.

Demographic and Medical Data—The owners’ questionnaires were reviewed for data regarding individual dogs, including signalment (age, sex and breed), vaccination (yes/no), deworming (yes/no), ownership (private household/breeding farm), supervised walks by the owners (yes/no), feeding (commercial only/commercial plus human food), raw food (yes/no), hospitalization within the previous year (yes/no), antibiotic treatment within the previous year (yes/no), participation in shows (yes/no), coprophagia of any other animal species (including other dogs, cats, horses, etc., yes/no) and the presence of additional animals within the same household (yes/no).

### 4.2. Bacterial Isolation and Species Identification

The rectal specimens [14] were collected using bacteriological swabs (Meus s.r.l., Piove di Sacco, Italy) and were inoculated directly into a Luria Bertani infusion enrichment broth (Hy-Labs, Rehovot, Israel) to increase the sensitivity of the ESBL-PE detection [15]. After incubation at 37 °C (18–24 h), the enriched samples were plated onto CHROMagarESBL plates (Hy-Labs, Rehovot, Israel), at 37 °C for 24 h. The colonies that appeared after overnight incubation at 37 °C were recorded, and one colony of each distinct color and morphology was re-streaked onto a fresh CHROMagarESBL plate (Hy-Labs, Rehovot, Israel) to obtain a pure culture. The pure isolates were stored at −80 °C for further analysis.

The isolates were subjected to Vitek-MS (BioMérieux, Inc., Marcy-l’Etoile, France) for species identification or to Vitek-2 (BioMérieux, Inc., Marcy-l’Etoile, France) for species identification and/or antibiotic susceptibility testing (AST-N270 Vitek 2 card (BioMérieux, Inc., Marcy-l’Etoile, France)). The ESBL-production was confirmed by a combination of disk diffusions using cefotaxime and ceftazidime discs (Oxoid, Basingstoke, UK), as well as cefotaxime and ceftazidime with clavulanic acid (Sensi-Discs BD, Breda, The Netherlands). The results were interpreted according to the Clinical and Laboratory Standards Institute (CLSI) guidelines [16]. A bacterium was considered as ‘resistant’ to a certain antibiotic only if a full resistance result was determined (an ‘intermediate’ result was considered as ‘susceptible’). The MDR bacteria were defined as such due to an in vitro resistance to three or more classes of antimicrobial agents [17].

### 4.3. Sample Size and Statistical Analysis

The minimal sample size (number of animals sampled) was calculated using WinPepi, based on an estimated gut colonization rate of 15% [47], with a confidence level of 95% and an acceptable difference of 7.5%, resulting in *n* = 88. The risk assessment was performed using Chi-square or Fisher’s exact tests for association between the individual variables and ESBL-PE gut colonization. The descriptive statistics were used to describe the gut colonization rates. The continuous variables were analyzed using *t*-tests or Mann–Whitney U-tests. *p* ≤ 0.05 was considered statistically significant. For the risk factor analysis, a logistic regression model (multivariable analysis) was conducted using all of the significant variables in the univariable analysis at a significance level of *p* ≤ 0.2 using the ENTER method (IBM SPSS Statistics 25 (IBM, New York, NY, USA)).

### 4.4. Whole Genome Sequencing

Ten isolates, which were recognized as ESBL-PE, were subjected for whole genome sequencing (WGS). The DNA was purified using magLEAD (Precision System Science Co., LTD, Matsudo, Japan), according to the manufacturer’s instructions. Paired-end libraries were generated using an Illumina Nextera XT DNA library preparation kit, according to the Illumina protocols. For sequencing, we utilized the Illumina MiSeq platform using a 250-bp paired-end read kit v2. All bioinformatic analyses were performed using the PATRIC v3.5.36 platform [48] with default parameters, unless otherwise noted. The Fastq files were uploaded to the PATRIC server and analyzed by the Comprehensive Genome Analysis Service. The phylogenetic tree was generated using the PATRIC Phylogenetic Tree Building Service: the Codon Tree method selects single-copy PATRIC PGFams and analyzes 1000 aligned, shared (by all of the genomes) single-copy genes, using the program RAxML. As a reference, two NCBI representative genomes, which are considered to be of a high quality, were included in the phylogenetic tree. The mafft program was used for the alignment of 404,477 amino acids and 1,213,431 nucleotides. The best protein model found by RAxML was JTT. The read quality was assessed using FastQC v0.11.8 and MultiQC v1.7, and the taxonomic sequence classification of the *Enterobacterales* isolates was determined using KRAKEN2 (Table S1, Supplementary Materials). The multilocus sequence type (MLST) profile was determined using the MLST 2.0 server at the Center for Genomic Epidemiology, DTU, Research group for Genomic Epidemiology-National Food Institute-Technical University of Denmark [29] and PubMLST (Table 1) [49]. The antibiotic resistance genes were identified using the NCBI National Database of Antibiotic Resistant Organisms (NDARO) and the PATRIC v3.5.36 platform. In addition, the presence of the beta-lactamase genes was confirmed by the Beta Lactamase Data base [50]. The location of the antibiotic resistance genes on the contigs sequences was determined and the whole contig sequence was used to search the nr/nt nucleotide collection, using blastn (megablast, at the National Center for Biotechnology Information (NCBI) [51], to determine if the resistance genes were located on the chromosome or on a plasmid. For the comprehensive analysis of resistance genes, the genomes fasta files were uploaded to the Comprehensive Antibiotic Resistance Database (CARD), server available online [52] and the Resistance Gene Identifier (RGI) was used to predict bacterial resistome(s), based on homology and SNP models. The results of this analysis appear in Table S1 (Supplementary Materials). The mobile genetic elements (MGEs) were detected by uploading fasta files into the MobileElementFinder web tool at the Center for Genomic Epidemiology, available online [29].

### 4.5. Data Availability

The sequences are now available to the public at the NCBI (BioProject number PRJNA745537).

## 5. Conclusions

The results of this study reveal that healthy community dogs in Israel may be colonized with ESBL-PE MDR strains, similarly to the global prevalence of ESBL-P *E. coli*, and in a lower prevalence compared to other companion animals’ community cohorts, and the cattle population in the country. The WGS of the colonizing ESBL-PE bacteria indicate a non-clonality situation and reveal a wide arsenal of ARGs that may have environmental shedding potential. Further studies and active surveillance should investigate the ESBL-PE spread and transmission routes.

## Figures and Tables

**Table 1:** The ESBL-PE isolates colonizing healthy community dogs.

ESBL-PE Isolate	Bacterial Species	*E. coli* Phylogenetic Group	Sequence Type	Antimicrobial Resistance ^1,2^						
				AMC	AMK	GENT	NIT	CIP	TMS	IMP
C13.1	*E. coli*	A	46							
C17.1	*E. coli*	A	10							
C22.1	*E. coli*	A	10							
C23.2	*Citrobacter portucalensis*		165							
C117.1	*E. coli*	B1	10							
C129.1	*E. coli*	B1	297							
C129.2	*Citrobacter braakii*		109							
C136.1	*E. coli*	C	88							
C160.1	*E. coli*	B1	602							
C164.1	*E. coli*	B1	1586							

^1^ A black box indicates resistance, a white box indicates susceptibility; ^2^ AMC—amoxicillin clavulanate; AMK—amikacin; GENT—gentamicin; NIT—nitrofurantoin; CIP—ciprofloxacin; TMS—trimethoprim-sulfamethoxazole; IMP—imipenem.

**Table 2:** The resistome of the ESBL-PE isolates colonizing healthy community dogs, predicted by NDARO and the β-Lactamase Data base (BLDB). Plasmid replicons were detected by the MobileElementFinder web tool.

ESBL-P Isolate	*Bla* Genes	Quinolone	Tetracycline	Aminoglycoside	Macrolides	Sulfonamides	Trimethoprim	Chloramphenicol	Plasmid Replicon(s)	Plasmid/ Chromosome (Best Plasmid Homology by Blastn Search)	Notes
C13.1	PSE-1 CMY-171 TEM-156		Tet(A)	AadA2, CmlA1, AadA1	Mef(B)	Sul3			IncI1, IncR, ColpVC, Col(MG828), IncFIA(HI1)	pMRSN346355_67.9	
C17.1	*bla*CTX-M-3 AmpC								IncFIB(AP001918, IncFII(pSE11), Col(MG828)	pZ0117KP0004-1	Probably horizontal transfer from *Klebsiella pneumoniae* ^1^
C22.1	*bla*CTX-M-3 *bla*TEM-1 AmpC	QnrS1	Tet(A), Tet(B)	APH (3″)-Ib, APH (6)-Id		Sul2	DfrA14		IncFII(pHN7A8), IncY	pCFSAN061768	
C23.2	*bla*CMY-34	QnrB9								Chromosome	
C117.1	CTX-M-55 *bla*TEM-234 *bla*EC		Tet(A)	AAC (3)-Iia, APH (3′)-Ia, APH (3″)-Ib, APH(6)-Id	Mef(B)	Sul2	DfrA14	CatII	IncFIB(AP001918), IncFIC(FII)	pTREC1	
C129.1	*bla*CTX-M-100 TEM-234 *bla*TEM-156	QnrVC1	Tet(B), Tet(C)	AadA5, APH (3′)-Ia, AadA6	Mph(E)	Sul1, Sul2	DfrA17	CatA1	IncA/C2, IncY	pS44 related	Probably novel plasmid
C129.2	*bla*CTX-M-39 *bla*TEM-237 blaCMY-93	QnrVC1	Tet(B), Tet(C)	AadA6, APH(3″)-Ib, APH(6)-Id, APH(3′)-Ia	Mph(E)	Sul1		CatA1	IncA/C2	pS44 related	Probably novel plasmid
C136.1	SHV-12 AmpC	QnrB	Tet(A)						IncFIB(AP001918), IncFIC(FII), Col(MG828), IncX3, IncI1	pEC-147 related	
C160.1	*bla*CTX-M-55 *bla*EC *bla*TEM-243		Tet(A)	AadA1, APH (3″)-I, APH (6)-Id, Sul2		Sul2	DfrA1	FloR	IncFIB(AP001918), IncFIC(FII), Col(MG828), IncN	Chromosome, plasmid pAH01-3-related	bla genes are located on a plasmid
C164.1	blaCTX-M-3 *bla*TEM-237 blaEC-2199			APH (3″), AAC (3)-Ii, APH(6)-Id	Mph(E)	Sul1	DfrA12		IncL/M, IncFIB(pHCM2)	pA1-3	Probably horizontal transfer from *Klebsiella pneumoniae* ^2^

^1^ NCBI Reference Sequence: NZ_CP098168.1; ^2^ NCBI Reference Sequence: NZ_LC508263.1.

**Table 3:** Univariable analysis of ESBL-PE gut colonization by healthy community dogs.

Variable	Classification	No. (Valid%)	*p*-Value	OR (95% CI)
Age (years), median (range)	2.6 (one month—12.5)		0.179	
Sex	Female	86 (63.2)	>0.999	0.851 (0.203–3.563)
	Male	50 (36.8)		
Vaccination	Yes/No	143 (99.3)	>0.999	0.993 (0.978–1.007)
Deworming	Yes/No	115 (89.8)	0.568	0.778 (0.088–6.87)
Ownership	Private household	112 (77.2)	0.21	0.757 (0.689–0.833)
	Breeding farm	33 (22.8)		
Supervised walks by the owners	Yes/No	132 (91.7)	0.165	0.28 (0.051–1.53)
Commercial feeding only	Yes/No	107 (73.8)	>0.999	1.26 (0.25–6.348)
Raw food	Yes/No	9 (6.3)	>0.999	0.933 (0.891–0.976)
Hospitalization within the previous year	Yes/No	20 (14.3)	>0.999	0.85 (0.099–7.301)
Antibiotic treatment within the previous year	Yes/No	40 (29.2)	0.416	1.885 (0.402–8.834)
Participation in shows	Yes/No	5 (6.8)	>0.999	0.925 (0.865–0.99)
Coprophagia	Yes/No	44 (30.5)	0.024 *	5.105 (1.215–21.457)
The presence of additional animals within the same household	Yes/No	113 (78.5)	0.405	0.523 (0.123–2.224)

* Significant (*p* < 0.05).

**Table 4:** Multivariable analysis of ESBL-PE gut colonization by healthy dogs in Israel.

Variable	*p*-Value	OR (95% CI)
Age	0.149	0.762 (0.527–1.102)
Supervised walks	0.137	0.243 (0.038–1.569)
Coprophagia	0.048 *	4.408 (1.014–19.169)

* Significant (*p* < 0.05).

## Data Availability

The sequences are now available to the public at the NCBI (BioProject number PRJNA745537).

## Supplementary Materials

The following supporting information can be downloaded at: https://www.mdpi.com/article/10.3390/antibiotics11081069/s1, Table S1: A comprehensive analysis of all of the genes that may contribute to the isolate resistance was performed using CARD, by utilizing the Resistance Gene Identifier (RGI) function; Table S2: mobile genetic elements (MGEs), detected by the MobileElementFinder web tool.
